# Decomposition of functional beta, but not alpha, diversity detects deviations from the “host-diversity-begets-parasite-diversity” rule in flea-mammal associations

**DOI:** 10.1007/s00436-025-08527-8

**Published:** 2025-07-08

**Authors:** Boris R. Krasnov, Irina S. Khokhlova

**Affiliations:** 1 Mitrani Department of Desert Ecology, Swiss Institute for Dryland Environmental and Energy Research, Jacob Blaustein Institutes for Desert Research, Ben-Gurion University of the Negev, Sede Boqer Campus, Midreshet Ben-Gurion, Israel; 2 French Associates Institute for Agriculture and Biotechnology of Drylands, Jacob Blaustein Institutes for Desert Research, Ben-Gurion University of the Negev, Sede Boqer Campus, Midreshet Ben-Gurion, Israel

**Keywords:** Dissimilarity, Fleas, Functional diversity, Small mammals, Resemblance

## Abstract

The “host-diversity-begets-parasite-diversity” (HDBPD) pattern has been demonstrated for traditional metrics of functional diversity. We applied the decomposition of functional alpha (functional diversity structure; FDS) and beta (functional resemblance; FR) diversity into (a) Simpson’s dominance, functional redundancy, and functional diversity and (b) taxonomic similarity, functional dissimilarity, and beta redundancy, respectively, coupled with ternary diagrams, to test whether the HDBPD rule also holds for FDS and FR and their separate components for communities of fleas and small mammals from different biomes of the Palearctic. We compared patterns of FDS and FR and their components within and between fleas and hosts within and between biomes and tested for correlation of separate components of functional alpha and beta diversities between fleas and hosts. Differences in FR between biomes were detected for both fleas and hosts (due to differences in each FR component), but not in FDS. Within biomes, fleas and hosts demonstrated marginally significant differences in FDS in steppes and temperate forests due to differences in the functional diversity component. Differences in FR between fleas and hosts were found in four of five biomes (due to differences in one or two FR components). Values of each of the three separate components of the flea FDS correlated positively with values of the respective components of the host FDS suggesting the effect of functional alpha diversity of hosts on that of fleas. No correlation between separate components of the flea and host FR was found implying no link between host and flea functional beta diversity. We conclude that the HDBPD rule, when applied to functional beta diversity, is not as universal as it was previously thought to be.

**Supplementary Information:**

The online version contains supplementary material available at 10.1007/s00436-025-08527-8.

## Introduction

The diversity of any group of species is affected by a variety of both abiotic and biotic factors. The latter are especially important for consumer species since they ultimately depend on species that constitute their resources. One consequence of this dependence is the tight relationship between consumer and resource diversities that has been demonstrated in various consumer-resource associations (e.g., Siemann et al. 1998; Rezende et al. 2007; Nilsson et al. 2008; Jetz et al. 2009). Parasites strongly depend on their hosts since the latter provide parasites not only with food but also with a place to live, mate, and reproduce. Much evidence has demonstrated that host species richness is the main factor determining parasite species richness (Krasnov et al. 2004a, 2020; Hechinger and Lafferty 2005; Thielgles et al. 2011; Poulin 2014; Kamiya et al. 2014; Maestri et al. 2017; Eriksson et al. 2020). Therefore, one of the central rules in parasite ecology is “host diversity begets parasite diversity” (Hechinger and Lafferty 2005). Moreover, taxonomic dissimilarity between host assemblages (i.e., taxonomic host beta diversity) has been shown to be an important driver of taxonomic beta diversity of parasite assemblages (Krasnov et al. 2020; see also Henriksen et al. 2022).

It is commonly recognized that the diversity of living organisms includes not only the number and composition of taxonomic units, such as species (taxonomic diversity), but also encompasses the number and composition of phylogenetic units, such as lineages (phylogenetic diversity), and functional units, such as traits or trait complexes (functional diversity) (e.g., Faith 1992; Tilman et al. 1997; De Bello et al. 2010; Schmera et al. 2023). Strong relationships between host and parasite phylogenetic and functional diversities have also been shown (Clark et al. 2018; Krasnov et al. 2019, 2024), although some geographic variation in this pattern has been reported (e.g., Krasnov et al. 2024). One of the main mechanisms behind the link between parasite and host functional diversities is thought to be “trait matching” because host and parasite traits have coevolved in response to each other (Decaestecker et al. 2007; McQuaid and Britton 2013; Buckingham and Ashby 2022).

Similarly to taxonomic (= compositional) diversity, different levels of functional diversity are recognized, such as functional alpha diversity (functional diversity within a locality/habitat) and functional beta diversity (functional dissimilarity between localities/habitats) analogously to compositional alpha diversity and beta diversity proposed by Whittaker (1960, 1972). Functional alpha and beta diversity each represents a complex of several components. For example, functional alpha diversity can be partitioned into functional richness, functional divergence, and functional regularity (the variability of functional units) (Mason et al. 2005; Schmera et al. 2023), whereas replacement and richness differences were proposed as components of functional beta diversity (Cardoso et al. 2014). Earlier studies of functional alpha and beta diversities, partitioned into components, demonstrated that the behaviour of these components along spatial or environmental gradients (including either abiotic or biotic factors or both) might be different (Schumm et al. 2019; Krasnov et al. 2019, 2024; Maestri et al. 2020).

Recently, Ricotta et al. (2023) and Ricotta and Pavoine (2024) proposed novel methods to decompose functional diversity. In their approaches, functional alpha diversity is decomposed into (a) Simpson’s dominance D (a complement of Simpson’s diversity; i.e., the contribution to the mean similarity if two randomly selected individuals within a community belong to the same species); (b) functional redundancy R (the amount of species diversity not associated with functional diversity), and (c) Rao’s functional diversity Q (= Rao’s quadratic entropy; average pairwise trait-based species dissimilarity). Functional beta diversity is decomposed into (a) the standard taxonomic similarity in the relative abundances of species between sites S_BC_ (BC: after the Bray–Curtis similarity), (b) the degree of functional dissimilarity between distinct species between sites D_KG_ [(KG: after the dissimilarity coefficient of Kosman (1996) and Gregorius et al. (2003)], and (c) the degree of functional similarity between distinct species between sites, or beta redundancy R_β_. Taken together, the three components of functional alpha diversity represent the functional diversity structure (FDS) of a community, whereas the three components of functional beta diversity represent the functional resemblance (FR) between communities [see rationales, equations, and proofs in Ricotta et al. (2021, 2023) and Ricotta and Pavoine (2024)]. The advantage of both decompositions is that the three components of functional either alpha or beta diversity (a) vary from 0 to 1 and (b) are additive, with their sums being unity. This allows visualizing FDS or FR using a ternary diagram, which presents the values of the three diversity components as point coordinates on a triangle with equal angles and sides of the same length. Each community is represented by a point with a position determined by the values of the Q, R and D or D_KG,_ R_β_ and S_BC_. This allows to compare (a) taxonomic and functional diversity or (b) taxonomic and functional variability, respectively, between communities in more details than by using a single measure of functional diversity.

The only application of Ricotta et al.’s (2023) method of decomposing functional alpha diversity to parasites aimed at investigating the differences in the functional diversity structure between flea and gamasid mite communities (i.e., assemblages harboured by the same host species in the same locality at the same time; Bush et al. 1997) in different host species, biomes, continental sections, and habitats, and demonstrated significant differences in the values of D and R but not Q (Krasnov et al. 2025). This provided a more thorough understanding of the effects of hosts, environment, and geographic position on patterns of functional variation in parasites. In particular, it appeared that the functional diversity structure of flea and mite communities differed mainly between host species within a biome/habitat or geographic regions/locations rather than between biomes/habitats or geographic regions/locations. Comparison of these results with the results of earlier studies has led to conclusion that the DRQ approach had an advantage over a single diversity metric and allowed a better understanding of spatial variation in different facets of ectoparasite diversity. Decomposition of functional beta diversity (Ricotta and Pavoine 2024) to study patterns of FR in parasite communities has never been applied.

Although studies of parasite and host functional diversities, using traditional metrics, have suggested a strong link between them (see above), functional alpha and beta diversity decompositions, coupled with the ternary diagrams (Ricotta et al. 2023; Ricotta and Pavoine 2004), had never been applied to compare patterns of functional variation between parasites and hosts and to test for the association between their functional diversities. It remained unknown whether the “host-diversity-begets-parasite-diversity” rule also holds for FDS and FR and their separate components. To understand this, we studied FDS and FR in compound communities of fleas (i.e., assemblages of fleas harboured by all host species in a locality; Bush et al. 1997) and their small mammalian hosts from different biomes of the Palearctic, using the same sources of data as in Krasnov et al. (2025). We compared patterns of FDS and FR (overall and their separate components) (a) within fleas and hosts between biomes and (b) between fleas and hosts within a biome. Then, we tested for correlation of separate components of functional alpha and beta diversities between fleas and hosts.

## Materials and methods

### Data on flea and host distributions

We re-used the data on flea distribution in their small mammalian hosts (Rodentia, Soricidae, Talpidae and Ochotonidae) from Krasnov et al. (2025). In brief, these data were taken from published surveys that reported the number of flea individuals belonging to a particular species collected from a specified number of individuals of a particular host species from 45 regions of the central, northern, and western Palearctic. The lists of regions, details on sampling procedures, numbers of flea and host species, maps, and references were previously published (Krasnov et al. 2015; Warburton et al. 2017).

To calculate the relative abundances of fleas, we selected host species in which at least 10 individuals in a region were parasitologically examined. We then calculated the relative abundances of each flea species per region, using the number of flea individuals of a given species collected from all host species. The relative abundances of each host species in a region were calculated as proportions of individuals of this species across all individuals of all host species collected in this region, assuming that the number of examined hosts of each species reflected this species’ relative abundance (the data on independent estimations of host abundances were unavailable in the original sources). In total, we used data on 1,727,402 flea individuals belonging to 202 species and 541,218 host individuals belonging to 123 species.

### Flea and host traits

We characterized each flea species by five quantitative and three nominal traits. These traits were: (a) characteristic abundance on the principal host across a flea’s geographic range (controlled for unequal sampling effort); (b) the number of host species on which a flea was recorded across its geographic range (controlled for unequal sampling effort); (c) these hosts’ phylogenetic diversity; (d) body size; (e) the degree of sexual dimorphism; (f) microhabitat preference (i.e., whether most of the flea’s life is spent on the host’s body or in its burrow or nest, or both); (g) main reproductive season (summer, winter, or year-round); and (h) the number of sclerotized combs (ctenidia) used by fleas to anchor themselves in hosts’ hair, thus withstanding hosts’ grooming efforts (no combs, one comb, or two combs). The rationale, details of the calculations, measurements of these traits, and the sources of information on them can be found elsewhere (Krasnov et al. 2015, 2016, 2025). In particular, Krasnov et al. (2006) found that abundances of the same flea species on the same host species, but in different regions, were more similar to each other than expected by chance, and varied significantly among flea species. Abundance has thus been shown to be a true species trait in fleas that varies only within narrow species-specific boundaries. Similarly, the number of host species exploited by a flea species and taxonomic distinctness of these hosts (as a proxy of their phylogenetic diversity) have been found to vary significantly more among flea species than within flea species demonstrating that these metrics of host specificity are also true attributes (= traits) of a flea species (Krasnov et al. 2004b). Furthermore, the number of host species exploited by a flea and phylogenetic diversity of these hosts represent different facets of host specificity of the flea, namely (a) how many host species can successfully be used by this flea and (b) how closely related these host species are (Poulin et al. 2011). For example, imagine that two flea species exploit the same number of host species, but phylogenetic diversity of the hosts used by flea species A is higher than that of flea species B. Therefore, species B is characterised by higher phylogenetic host specificity because of host phylogenetic spectrum of its hosts is narrower. In other words, the ability of species A to exploit a variety of hosts is higher than that of species B.

Each host species was characterized by four quantitative, one ordinal, and five nominal traits, selected because flea parasitism presumably responds to variation in these host traits (Krasnov et al. 2004a, 2016; Krasnov 2008). These traits were (a) average body mass; (b) relative brain mass; (c) dispersal range (mean distance between the birth and the breeding locations); (d) geographic range size; (e) habitat breadth (the number of distinct level 1 IUCN habitats); (f) nest location (on, above, or below ground); (g) life style (ground-dwelling, fossorial, arboreal, or a combination of styles); (h) diel activity (diurnal, nocturnal, or cathemeral); (i) feeding habits (omnivory, folivory, granivory, insectivory, or a combination of feeding habits); and (j) hibernation/torpor pattern (hibernation/torpor or not). Data on host traits were obtained from the PanTHERIA database (Jones et al. 2009), the EltonTraits database (Wilman et al. 2014), and the COMBINE database (Soria et al. 2021).

For both fleas or hosts, data on quantitative traits were scaled between 0 and 1. Then, matrices of functional dissimilarities between flea or host species were constructed using the ‘‘dist.ktab” function from the ‘‘ade4″ package (Thioulouse et al. 2018), implemented in the R Statistical Environment (R Core Team 2024), and calculating Gower’s distances (Gower 1971) between flea or host species.

### Data analysis

We partitioned the regional data according to the predominant biome of a region [boreal forests (nine regions), deserts (five regions), mountains (13 regions), steppes (11 regions), and temperate forests (seven regions); see Warburton et al. 2017; Krasnov et al. 2025]. Data on the relative abundances of fleas and hosts, combined with the respective matrices of their functional distances, were used to calculate the FDS and FR components for flea and host assemblages in each region as follows. For FDS (decomposition of functional alpha diversity), Rao’s functional diversity Q was calculated using the “QE” function of the R package “adiv” (Pavoine 2020). Then, Simpson’s diversity (S) was calculated using the “speciesdiv” function of the “adiv” package with option method = “GiniSimpson”, and Simpson’s dominance D and functional redundancy R were calculated as D = 1 – S and R = S – Q, respectively (see Ricotta et al. 2023). For FR (decomposition of functional beta diversity), we calculated the mean (for a region) values of functional dissimilarity (D_KG_), beta redundancy (R_β_), and the Bray–Curtis taxonomic similarity (S_BC_), relative to all other regions characterized by the same predominant biome (see Ricotta et al. 2021). This was done using the “betaUniqueness” function of the R package “adiv” with the R code of Ricotta and Pavoine (2024).

To visualize differences in FDS or FR (a) within fleas or hosts between biomes and (b) between fleas and hosts within biomes, we plotted the mean values of either D, R, and Q, or D_KG_, R_β_, and S_BC,_ respectively, on ternary diagrams. Ternary diagrams were generated using the R package “Ternary” (Smith 2017). Then, distance-based multivariate analyses of variance (db_MANOVAs), implemented in the R package “PERMANOVA” (Vicente-Gonzalez and Vicente-Villardon 2021), were used to test the above-mentioned differences in FDS or FR. Db_MANOVA is a non-parametric multivariate generalization of a traditional ANOVA that compares the within-group versus the between-group dissimilarities (Anderson 2001), allowing one to test the differences between two or more groups of assemblages. Pairwise dissimilarities in FDS (i.e., the DRQ combination) or FR (i.e., the D_KG_R_β_S_BC_ combination) within fleas or hosts between biomes, as well as between fleas and hosts within each biome, were calculated using the Bray–Curtis dissimilarity, and the *p*-values were obtained by 10,000 random permutations.

Because db_MANOVAs cannot detect which particular component of FDS (D, R, or Q) or FR (D_KG,_ R_β_, or S_BC_) differs between groups (within fleas or hosts between biomes or between fleas and hosts within a biome), we followed Ricotta et al. (2023) and Ricotta and Pavoine (2024) and further tested for the above-mentioned differences in each FDS or FR component using univariate ANOVAs with 10,000 random permutations of flea or host assemblages between the respective groups. This was done using the R package “RRPP” (Collyer and Adams 2018). Finally, we analysed the effect of each separate component of decomposed host functional alpha or beta diversity on the respective component of decomposed flea functional alpha or beta diversity. Because the components of the FDS or FR vary between 0 and 1 but do not attain these extreme values, we applied beta regression using the R package “betareg” (Cribari-Neto and Zeileis 2010).

## Results

### Functional alpha diversity

**Fig. 1:**
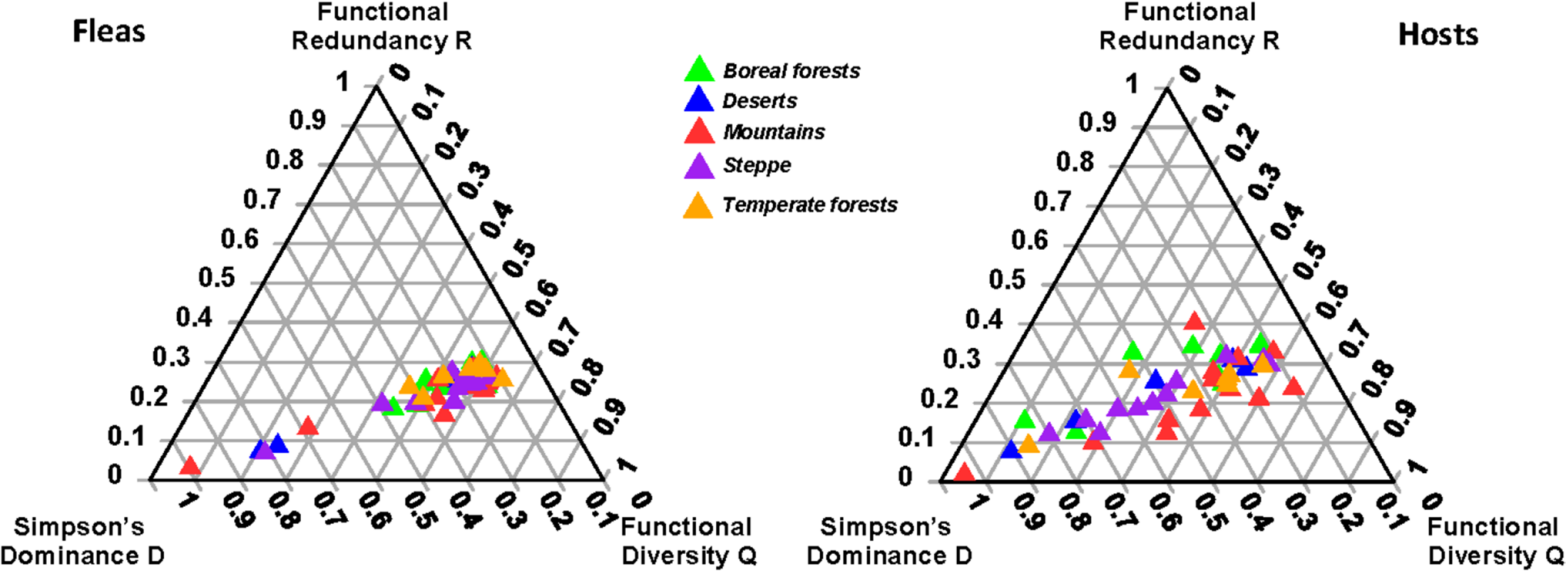
The ternary diagrams of functional diversity structure (FDS; functional alpha diversity) for regional flea and host assemblages in different biomes

The results of distance-based multivariate analyses of variance (db-MANOVAs) demonstrated that the FDS of regional assemblages (represented by a combination of the three components; see above) did not differ significantly between-biomes either in fleas (proportion of variance explained = 0.08, *F* = 0.93, *p* = 0.45) or in hosts (proportion of variance explained = 0.04, *F* = 0.44, *p* = 0.80) (Fig. 1). The same was true for the separate D, R, and Q components of the FDS as revealed by permutational univariate ANOVAs (Table 1; see mean values in Supplementary Table S1).

**Table 1:** Summary of permutational univariate ANOVAs testing for differences in the DRQ values of FDS (D: Simpson’s dominance; R: functional redundancy; Q: Rao’s quadratic diversity) between flea or host assemblages in dependence on the predominant biome of a region. All *p*-values were not significant

Fleas or Hosts	D/R/Q	*R*^*2*^	*F*	*p*
Fleas	D	0.08	0.92	0.46
	R	0.13	1.53	0.21
	Q	0.07	0.74	0.57
Hosts	D	0.04	0.40	0.81
	R	0.07	0.78	0.55
	Q	0.06	0.65	0.63

**Fig. 2:**
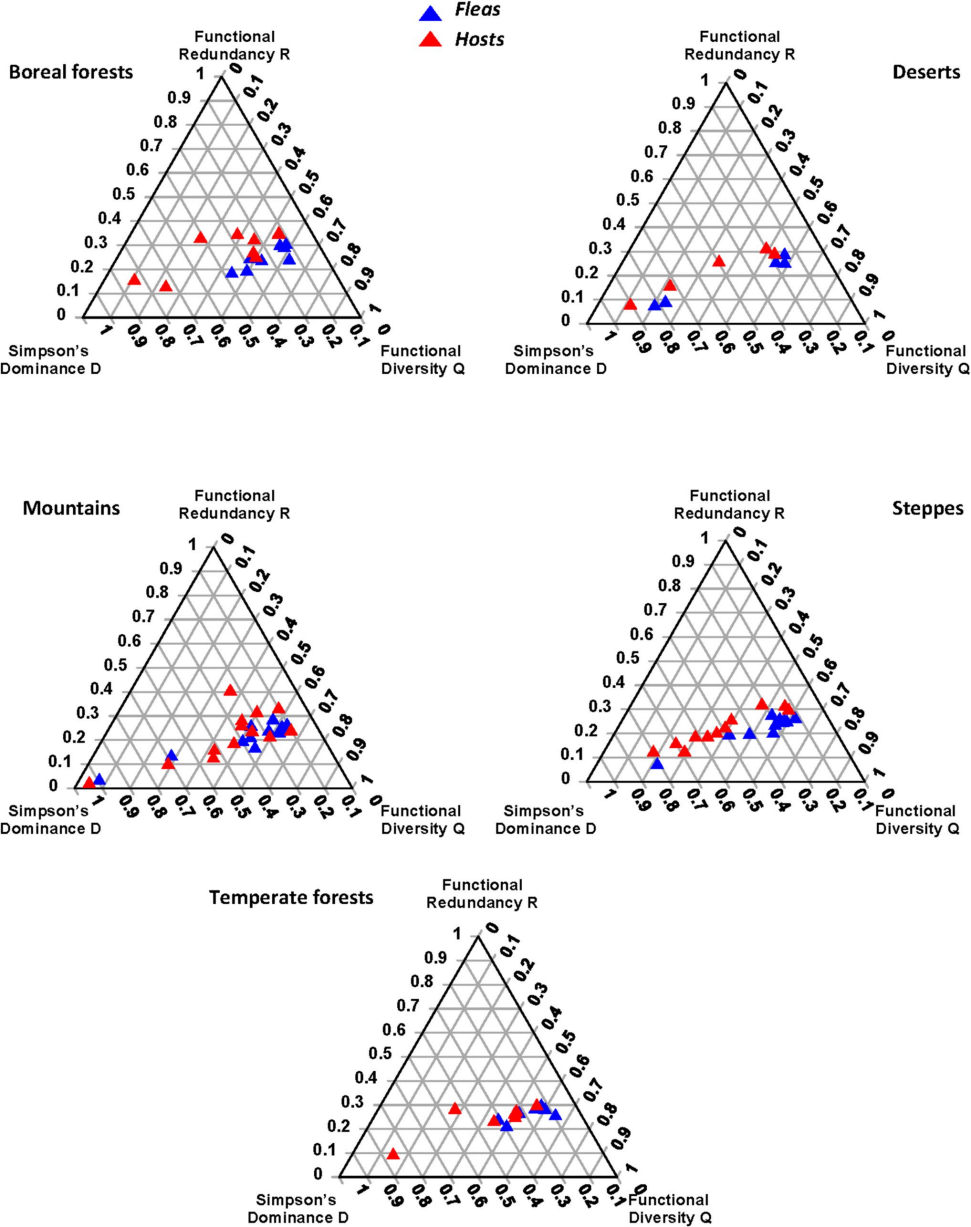
The ternary diagrams of functional diversity structure (FDS; functional alpha diversity) for flea and host assemblages from different biomes

Within biomes, flea and host assemblages demonstrated marginally significant differences in the FDS in steppes and temperate forests (Table 2, Fig. 2). This was mainly due to significant differences in the Q component of the FDS (measured as Rao’s quadratic diversity) (Table 3; see mean values in Supplementary Table S1). In addition, a significant difference between flea and host assemblages in boreal forests was detected for the Q component, but this was not further translated into a difference in an overall FDS.

**Table 2:** Summary of distance-based multivariate analyses of variance (db-MANOVAs) testing for differences in functional diversity structure (FDS; the DRQ composition of functional alpha diversity; see text for explanation) between flea and host assemblages from regions characterized by different predominant biomes. Marginally significant *p*-values are in bold font

Biome	Proportion of variance explained	*F*	*p*
Boreal forests	0.15	2.93	0.10
Deserts	0.02	0.15	0.65
Mountains	0.03	0.70	0.42
Steppes	0.17	4.09	0.06
Temperate forests	0.21	3.17	0.07

**Table 3:** Summary of permutational univariate ANOVAs testing for differences in the DRQ values of FDS (D: Simpson’s dominance; R: functional redundancy; Q: Rao’s quadratic diversity) between flea and host assemblages from regions characterized by different predominant biomes. Significant and marginally significant *p*-values are in bold font

PB/CS	D/R/Q	*R*^*2*^	*F*	*p*
Boreal forests	D	0.12	2.13	0.16
	R	0.04	0.77	0.39
	Q	0.30	6.92	**0.02**
Deserts	D	0.01	0.12	0.74
	R	0.02	0.19	0.67
	Q	0.07	0.59	0.46
Mountains	D	0.01	0.48	0.50
	R	0.01	0.07	0.79
	Q	0.04	1.28	0.27
Steppes	D	0.16	3.91	**0.06**
	R	0.01	0.03	0.85
	Q	0.29	8.37	**0.01**
Temperate forests	D	0.21	3.17	0.10
	R	0.04	0.51	0.49
	Q	0.27	4.45	**0.03**

Beta regression analyses demonstrated that values of each of the three separate components of the flea FDS correlated positively with values of the respective components of the host FDS (*p* = 0.0001–0.04) (Table 4).

**Table 4:** Summary of beta regressions of the effect of separate components of functional host alpha diversity (functional diversity structure FDS; D, R, Q components for host; see text for explanations) on the respective components of functional flea alpha diversity

Component	Coefficient	*z* value	*p*	Pseudo-R^2^
D	2.35 ± 0.47	5.01	< 0.001	0.36
R	2.72 ± 0.64	4.20	< 0.001	0.28
Q	2.00 ± 0.48	4.14	< 0.001	0.26

### Functional beta diversity

**Fig. 3:**
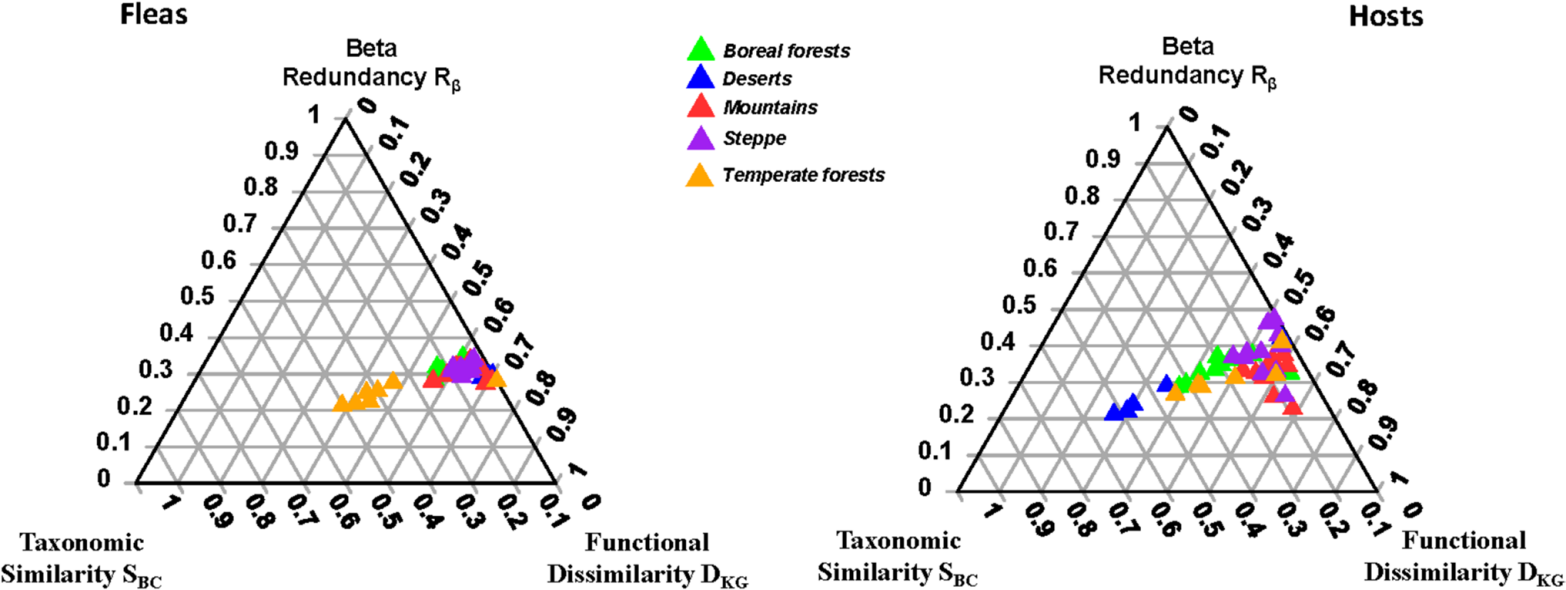
The ternary diagrams of functional resemblance (FR; functional beta diversity) regional flea and host assemblages in different biomes

In contrast to functional alpha diversity, functional beta diversity (FR) of flea and host assemblages differed significantly between biomes (proportion of variance explained = 0.64, *F* = 17.99 and proportion of variance explained = 0.49, *F* = 9.61, respectively; *p* < 0.001 for both) (Fig. 3). For example, the ternary diagram demonstrated differences in the FR between fleas from temperate forests or hosts from deserts and fleas or hosts from the remaining biomes (Fig. 3). The between-biome differences in the FR in fleas and hosts were due to differences in each separate component (D_KG,_ R_β_, and S_BC_) (Table 5; see mean values in Supplementary Table S2).

**Table 5:** Summary of permutational univariate ANOVAs testing for differences in the D_KG_R_β_S_BC_ values of FR (D_KG_: functional dissimilarity; R_β_: beta redundancy; S_BC_: taxonomic similarity) between flea or host assemblages in dependence on the predominant biome of a region. Significant *p*-values are in bold font

Fleas or Hosts	D_KG/_R_β/_S_BC_	*R*^*2*^	*F*	*p*
Fleas	D_KG_	0.58	13.68	** < 0.001**
	R_β_	0.65	18.34	** < 0.001**
	S_BC_	0.65	18.60	** < 0.001**
Hosts	D_KG_	0.55	12.22	** < 0.001**
	R_β_	0.29	4.11	**0.01**
	S_BC_	0.51	10.58	** < 0.001**

**Fig. 4:**
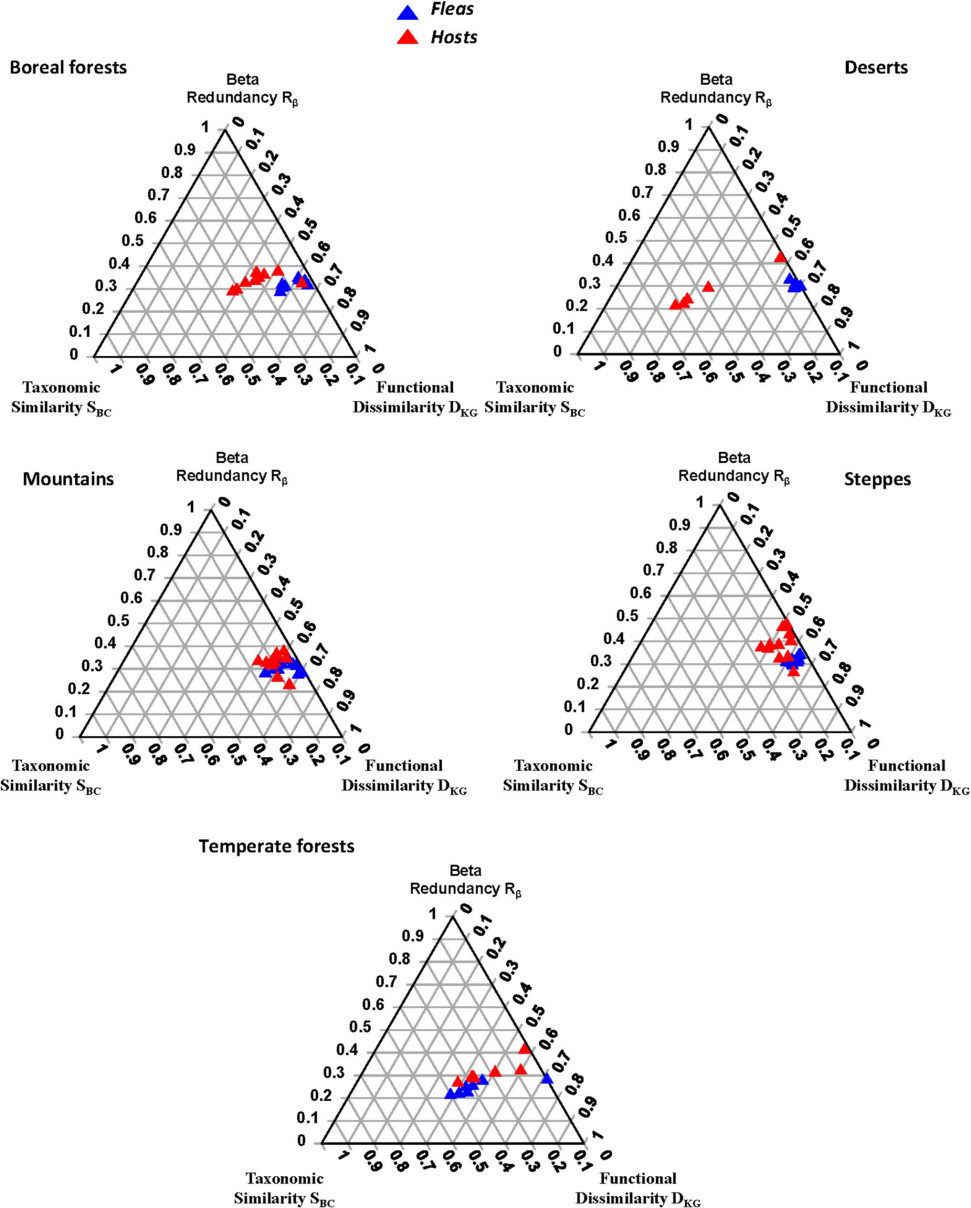
The ternary diagrams of functional resemblance (FR; functional beta diversity) for flea and host assemblages from different biomes

Differences in the FR between fleas and hosts were found in four of five biomes (except temperate forests) (Table 6; Fig. 4). In all biomes, flea and host assemblages significantly differed in either one or two of the three FR components (Table 7; see mean values in Supplementary Table S2). However, these differences resulted in differences in the overall FR in boreal forests, deserts, mountains, and steppes, whereas fleas and hosts from temperate forests differed in the beta redundancy component (R_β_); this, however, was apparently not enough to result in an overall FR difference.

**Table 6:** Summary of distance-based multivariate analyses of variance (db-MANOVAs) testing for differences in functional resemblance (FR; the D_KG_R_β_S_BC_ composition of functional beta diversity; see text for explanation) between flea and host assemblages from regions characterized by different predominant biomes. Significant *p*-values are in bold font

Biome	Proportion of variance explained	*F*	*p*
Boreal forests	0.49	15.68	** < 0.001**
Deserts	0.65	14.55	**0.01**
Mountains	0.13	3.68	**0.04**
Steppes	0.39	13.02	** < 0.001**
Temperate forests	0.11	1.48	0.22

**Table 7:** Summary of permutational univariate ANOVAs testing for differences in the D_KG_R_β_S_BC_ values of FR (D_KG_: functional dissimilarity; R_β_: beta redundancy; S_BC_: taxonomic similarity) between flea and host assemblages from regions characterized by different predominant biomes. Significant *p*-values are in bold font

Biome	D_KG/_R_β/_S_BC_	*R*^*2*^	*F*	*p*
Boreal forests	D_KG_	0.62	25.74	** < 0.001**
	R_β_	0.10	1.84	0.19
	S_BC_	0.45	13.35	** < 0.001**
Deserts	D_KG_	0.81	35.36	** < 0.001**
	R_β_	0.05	0.44	0.53
	S_BC_	0.64	14.35	**0.01**
Mountains	D_KG_	0.20	6.05	**0.02**
	R_β_	0.10	2.76	0.11
	S_BC_	0.06	1.68	0.21
Steppes	D_KG_	0.57	26.72	** < 0.001**
	R_β_	0.37	11.81	** < 0.001**
	S_BC_	0.05	1.18	0.29
Temperate forests	D_KG_	0.01	0.02	0.90
	R_β_	0.46	10.37	**0.01**
	S_BC_	0.09	1.17	0.30

The results of beta-regression analyses of separate components of the flea and host FR are presented in Table 8. No correlation between flea and host D_KG_, R_β_, or S_BC_ was found (*p* = 0.26–0.36).

**Table 8:** Summary of beta regressions of the effect of separate components of functional host beta diversity (functional resemblance FR; D_KG,_ R_β,_ S_BC_ components) on the respective components of functional flea beta diversity

Component	Coefficient	*z* value	*p*	Pseudo-R^2^
D_KG_	- 0.06 ± 0.49	−0.013	0.99	0.0001
R_β_	0.50 ± 0.37	1.33	0.18	0.04
S_BC_	−0.58 ± 1.00	−0.57	0.57	0.01

## Discussion

We compared patterns of functional alpha (FDS) and beta (FR) diversities within fleas and hosts between biomes as well as between fleas and hosts within a biome. We found between-biome differences mainly for functional beta, but not alpha, diversity.. Furthermore, functional beta, but not alpha, diversity appeared to differ between fleas and hosts within majority of biomes.

A positive relationship between parasite and host functional diversities has previously been reported for several parasite-host associations, such as gamasid mites and small mammals (Krasnov et al. 2019), helminths and small mammals (Cardoso et al. 2021), helminths and anurans (Euclydes et al. 2021), and fleas and small mammals (Krasnov et al. 2024). The most likely mechanism underlying this pattern is assumed to be the coevolution of parasite and host traits (McQuaid and Britton 2013), so that parasite traits track host traits (Poulin 2021). Although parasite-host coevolution, including coevolution of traits, is indubitable and, thus, the link between their functional diversities is not surprising, this relationship appears to be environmentally and geographically variable. This was revealed after partitioning functional diversity into its components. For example, when the functional alpha diversities of fleas and small mammals were partitioned into functional richness, regularity, and divergence, the positive correlations between flea and host functional richness and regularity appeared to be geographically invariant, whereas flea functional divergence correlated positively with host functional divergence in the Nearctic communities only (Krasnov et al. 2024). This suggests differential environmental, geographic, and host-associated effects on different parasite diversity components. Our study supported the strong link between functional alpha diversities of fleas and hosts. Moreover, the values of functional alpha diversity measured as a combination of species dominance, functional diversity, and functional redundancy did not vary between biomes. Therefore, different components of functional alpha diversity may or may not vary environmentally or geographically in dependence on the approach of the functional diversity decomposition.

In contrast to the functional alpha diversities of fleas and their hosts, the decomposition of the functional beta diversities, carried out in this study, revealed environmental variation in its separate components. Moreover, this decomposition demonstrated that the “host-diversity-begets-parasite-diversity” rule, when applied to functional beta diversity, is not as universal as it was earlier thought to be. The most likely explanation for the lack of a relationship between flea and host functional beta diversities is that flea and host assemblages might differ in the distribution of functional units (traits or trait complexes). In other words, host species in a community can be more functionally similar or dissimilar to each other than flea species are to each other or vice versa. For example, the values of functional dissimilarity D_KG_ differed between flea and host assemblages in deserts, but not in temperate forests (Fig. 4; Supplementary Material, Table S2). As a result, the FR differed between fleas and hosts in the former, but not in the latter, biome. Another, not necessarily alternative, explanation for the absence of a link between flea and host functional diversities can be associated with the phylogenetic composition of flea and host assemblages in a biome or a continental section because parasite and host trait complementarity is shaped by evolutionary history. Assuming that trait complementarity reflects phylogenetic congruence, it can be hypothesized that the variation in the degree of phylogenetic congruence (Dávalos et al. 2012; Sweet et al. 2018; Blasco-Costa et al. 2021), across different parasite and host assemblages, might lead to variation in the degree of trait complementarity and result in the presence or absence of the positive relationship between parasite and host functional diversities. We recognize, however, that this explanation is highly speculative and requires further investigation.

No relationship between flea and host functional beta diversities might result from differential environmental filtering of flea and host community assembly. Environmental filtering is a process in which the environment acts as a “filter”, allowing a community to contain only species possessing certain traits that are necessary to persist in that environment (Ackerly and Cornwell 2007; Ingram and Shurin 2009). It is possible that a given environment filters fleas and hosts differently, allowing a more homogeneous distribution of functional units among, for example, flea species, as compared to host species or vice versa. Air temperature can be an example of such a filter because of the very low variation between flea species in sensitivity to extreme temperatures (Krasnov 2008), whereas this is not the case for small mammals (e.g., Bozinovic et al. 2014). Another reason for the absence of the link between flea and host functional beta diversities can be associated with the effects of dispersal limitations, which are obviously more important for fleas than for hosts since fleas are unable to disperse on their own, instead using hosts as dispersal vehicles. These differences might result in the already mentioned differences in functional unit distributions because some flea species and, consequently, trait complexes might be lost during host dispersal, especially across natural barriers such as mountain ridges (e.g., Gibert et al. 2021).

The decomposition of functional diversity has been shown to result in the possibility of relating separate components to various processes affecting the structure of biological communities (Podani et al. 2013; Ricotta et al. 2023; Ricotta and Pavoine 2024). In our study, the decomposition of functional diversity into its elements allowed a better understanding of the role played by different diversity components in the spatial variation of functional alpha and beta diversities of compound flea communities and their respective host assemblages. In particular, the functional alpha diversity (FDS) of fleas and hosts was relatively stable from ecological perspective (Table 1). This was likely due to somewhat similar contributions of Simpson’s dominance, functional redundancy, and functional diversity to the overall flea and host FDS and the relatively low variation of these FDS components between biomes (Supplementary Material, Table S1). However, the functional beta diversity (FR) of fleas and host was spatially variable (Table 1) mainly due to variation in compositional turnover (S_BC_) (Supplementary Material, Table S2). Earlier, Krasnov et al. (2025) reported that the FDS of component flea communities varied mainly between host species within, but not between, biomes or geographic regions. Taken together, the results of Krasnov et al. (2025) and of this study suggest that different levels of functional diversity (alpha and beta) are governed by different rules.

A caveat regarding the definition of a trait should be mentioned. Violle et al. (2007) argued that functional traits are morphological and physiological attributes that may indirectly impact an organism’s fitness, thus emphasizing the link between functional traits and individual performance. According to this definition, a trait must be measured at the individual level. Later, Poulin (2021) modified this definition by including ecological features in addition to morphological and physiological ones, and by stating that functional traits might affect not only the fitness of an organism but also the success of a species. Therefore, a functional trait can characterize not only an individual, but also a species (e.g., geographic range size; see Poulin 2021 for additional examples). Our study implied the broad definition of Poulin (2021) rather than the narrow definition of Violle et al. (2007). Furthermore, data on individual- and species-based traits of small mammals are available, and comprehensive databases exist (see citations above). This, however, is not the case for fleas nor for many other parasite taxa, and trait databases are not available. In our study, the only flea trait measured in individuals is body size, whereas the remaining traits represent species characteristics that were collected from an enormous number of sources, including old and so-called “gray” publications. Consequently, the functional ecology of parasites, including fleas, warrants further data collections and investigations.

Although functional diversities of both hosts and parasites are driven, to a great extent, by their coevolutionary interactions that exert strong selective forces on their trait evolution (e.g., Buckingham and Ashby 2022), so that host and parasite functional diversities influence the dynamics of their relationships (e.g., Cardoso et al. 2021; Sun et al. 2023), the results of our study should be interpreted with some caution. This is because trait distributions differed between hosts and parasites. Although we selected those host traits that presumably affect flea parasitism and those flea traits that are presumably associated with their host selection, metrics of functional diversity might reflect somewhat different aspects of functional diversity when applied to different trait distributions. This, however, has never been specifically studied and requires further efforts. Moreover, the hypothetical lack of comparability between host and parasite functional diversities does not preclude visualizing their diversity components in the same ternary diagram. This is because both alpha and beta diversity components of the framework used here represent standardized metrics (Ricotta et al. 2023; Ricotta and Pavoine 2024).

The deviation of functional beta diversity from the “host-diversity-begets-parasite-diversity” rule has not been explicitly shown when earlier methods of functional beta diversity partitioning were used (e.g., Krasnov et al. 2024). This is most likely due to the fact that the approaches to decomposing functional beta diversity, proposed by Ricotta and Pavoine (2024) and implemented here, represent a more comprehensive view of functional diversity through combining different facets of functional species differences and similarities between communities, namely (a) the richness and abundance of distinct species (taxonomic diversity) and (b) ecological differences/similarities between species (functional diversity) (Gregorius and Kosman 2017; Ricotta et al. 2023). The latter is especially important because of the effects of these differences/similarities on the ecosystem or community functioning (Grime 1998; Naeem 1998). For example, under drastic environmental changes, (a) functionally similar species can replace each other, and/or (b) some of the sharply dissimilar species may easily adapt to new conditions, and thus, both categories would contribute to ecosystem stability (Ricotta et al. 2023). Therefore, the decomposition of functional beta diversity into the components proposed by Ricotta and Pavoine (2024) is a useful method, allowing the revealing of patterns that have not previously been envisaged when traditional diversity metrics or other partitioning methods were applied.

## Supplementary Information

Below is the link to the electronic supplementary material.

Supplementary file1 (DOCX 21 KB)

## Data Availability

Data on flea and host distribution can be found in the original sources and are deposited in the Dryad depository (Hadfield et al. 2013).
