# Supplementary material for: Decomposition of functional beta, but not alpha, diversity detects deviations from the “host-diversity-begets-parasite-diversity” rule in flea-mammal associations
Source: Parasitol Res. 2025 Jul 8;124(7):80. doi: 10.1007/s00436-025-08527-8 (PMC12238161; doi:10.1007/s00436-025-08527-8)
Supplement: Supplementary file 1 — Supplementary file1 (DOCX 21 KB) [file 436_2025_8527_MOESM1_ESM.docx]

**Supplementary Tables**

**Table S1.** Mean (±SE) values of Simpson’s dominance D, functional redundancy R and Rao’s quadratic functional diversity Q for flea and host assemblages from regions characterized by different predominant biomes

| Fleas or Hosts | Biome | D | R | Q |
| --- | --- | --- | --- | --- |
| Fleas | Boreal forests | 0.22±0.03 | 0.25±0.01 | 0.53±0.02 |
|  | Deserts | 0.38±0.13 | 0.19±0.04 | 0.43±0.08 |
|  | Mountains | 0.27±0.06 | 0.21±0.02 | 0.52±0.04 |
|  | Steppes | 0.26±0.05 | 0.22±0.02 | 0.52±0.03 |
|  | Temperate forests | 0.19±0.03 | 0.26±0.01 | 0.55±0.02 |
| Hosts | Boreal forests | 0.34±0.07 | 0.27±0.03 | 0.39±0.05 |
|  | Deserts | 0.44±0.12 | 0.22±0.04 | 0.34±0.08 |
|  | Mountains | 0.33±0.06 | 0.22±0.03 | 0.45±0.04 |
|  | Steppes | 0.41±0.06 | 0.21±0.02 | 0.37±0.04 |
|  | Temperate forests | 0.34±0.08 | 0.24±0.03 | 0.42±0.06 |

**Table S2.** Mean (±SE) values of functional dissimilarity D_KG_, beta redundancy R_β_ and taxonomic similarity S_BC_ for flea and host assemblages from regions characterized by different predominant biomes

| Fleas or Hosts | Biome | D_KG_ | R_β_ | S_BC_ |
| --- | --- | --- | --- | --- |
| Fleas | Boreal forests | 0.60±0.01 | 0.32±0.01 | 0.08±0.02 |
|  | Deserts | 0.68±0.01 | 0.30±0.01 | 0.02±0.005 |
|  | Mountains | 0.62±0.01 | 0.30±0.01 | 0.07±0.01 |
|  | Steppes | 0.62±0.004 | 0.31±0.004 | 0.06±0.01 |
|  | Temperate forests | 0.47±0.04 | 0.24±0.01 | 0.28±0.05 |
| Hosts | Boreal forests | 0.46±0.02 | 0.33±0.01 | 0.20±0.03 |
|  | Deserts | 0.35±0.05 | 0.28±0.04 | 0.37±0.09 |
|  | Mountains | 0.58±0.01 | 0.32±0.01 | 0.09±0.01 |
|  | Steppes | 0.54±0.01 | 0.38±0.02 | 0.08±0.01 |
|  | Temperate forests | 0.48±0.03 | 0.31±0.02 | 0.21±0.04 |
